# Increased Upper Trapezius Muscle Stiffness in Overhead Athletes with Rotator Cuff Tendinopathy

**DOI:** 10.1371/journal.pone.0155187

**Published:** 2016-05-09

**Authors:** Hio Teng Leong, François Hug, Siu Ngor Fu

**Affiliations:** 1 Department of Rehabilitation Sciences, The Hong Kong Polytechnic University, Hong Kong (SAR), China; 2 Laboratory “Movement, Interactions, Performance” (EA 4334), UFR STAPS, University of Nantes, France; 3 NHMRC Centre of Clinical Research Excellence in Spinal Pain, Injury and Health, School of Health and Rehabilitation Science, University of Queensland, Brisbane, Australia; Universidad Europea de Madrid, SPAIN

## Abstract

Although excessive tension of the upper trapezius (UT) is thought to contribute to rotator cuff tendinopathy, no study examined UT tension in athletes with and without rotator cuff tendinopathy. Here we used UT shear modulus measured using ultrasound shear wave elastography as an index of muscle stiffness/tension. The aims of this study were twofold: 1) to determine whether the UT muscle shear modulus is altered in athletes with rotator cuff tendinopathy compared to asymptomatic athletes, and 2) to detect optimal cut-off points of UT shear modulus in identifying athletes with rotator cuff tendinopathy. Forty-three male volleyball players (17 asymptomatic and 26 with rotator cuff tendinopathy, mean age = 22.9±3.5 years) participated in the study. UT shear modulus was quantified during active arm holding at 30° and 60° of shoulder abduction and passive arm positioning at 0°, 30° and 60° of shoulder abduction. During the active tasks, the UT shear modulus was higher in athletes with rotator cuff tendinopathy than the asymptomatic athletes (p = 0.002), regardless the arm position. During the passive tasks, athletes with rotator cuff tendinopathy exhibited a higher UT shear modulus than asymptomatic athletes only at 0° of shoulder abduction (13.0±2.5 kPa vs 10.2±1.8 kPa, p = 0.001). When considering the active task, an optimal cut-off shear modulus of 12.0 kPa at 30° of shoulder abduction (sensitivity = 0.84, specificity = 0.57, AUC = 0.757, p = 0.008) and 9.5 kPa at 60° of shoulder abduction (sensitivity = 0.88, specificity = 0.67, AUC = 0.816, p = 0.002) was detected. When considering the passive task at 0° of shoulder abduction, a cut-off of 12.2 kPa was found (sensitivity = 0.73, AUC = 0.817, p = 0.001). Findings from the present study show that monitoring passive and active UT muscle shear modulus may provide important information for the prevention/rehabilitation of rotator cuff tendinopathy.

## Introduction

Rotator cuff tendinopathy is considered to be the principal cause of shoulder pain in orthopedics and sports medicine [1,2], particularly in athletes with repetitive overhead activities [3]. Rotator cuff tendinopathy is defined as a mechanical entrapment of the subacromial soft tissues underneath the acromial arch during arm elevation [4,5].

Various muscles are responsible for the dynamic stability of the shoulder joint during arm movement. Among them, the upper trapezius (UT) muscle contributes to normal scapular motion by elevating and rotating the scapula during arm elevation [6,7]. Electromyography (EMG) studies highlighted the role of UT during shoulder abduction to preserve adequate kinematics of the glenohumeral joint and to stabilize the scapula [8–10]. However, excessive force produced by the UT might alter the normal kinematics of scapular motion and therefore contribute to rotator cuff tendinopathy [11–12]. To support this assumption, increased UT EMG amplitude was reported in people with rotator cuff tendinopathy compared to healthy individuals [13–15]. Although EMG provides important information about the neural control of movement it does not provide direct quantification of muscle tension or force [16], which is ultimately the most important information to understand the biomechanical effect of increased UT activation. The inability to accurately estimate the change in muscle force from the amplitude of surface EMG is explained by the fact the EMG signal is influenced by many physiological and non-physiological factors [17]. More importantly, EMG does not account for passive forces [16,18]. A more direct assessment of the mechanical behavior of the UT muscle is therefore important to improve risk identification/diagnosis, and in parallel, lay the foundation for the development of innovative and individualized preventive/rehabilitation strategies for people with rotator cuff tendinopathy.

Supersonic shear imaging (SSI) is a non-invasive ultrasound elastography technique to quantify muscle shear modulus by measuring the propagation velocity of the shear waves generated by acoustic radiation force [19–21]. Muscle shear modulus measured using SSI provides an accurate measurement of muscle stiffness and is linearly related to both active [22,23] and passive muscle force [24]. As a result, changes in muscle shear modulus measured during isometric tasks can be used to estimate changes in individual muscle tension [18]. Our previous findings indicated that the SSI technique provides a reliable (intra- and inter-operator) quantification of the UT shear modulus [25]. Therefore, SSI provides an opportunity to quantify change in UT muscle stiffness/tension during active and passive arm abductions in people with rotator cuff tendinopathy.

Here we used UT shear modulus measured using SSI as an index of muscle stiffness/tension [18]. The primary aim of the present study was to determine whether UT shear modulus measured during active arm holding and passive arm positioning is altered in athletes with rotator cuff tendinopathy compared to asymptomatic athletes. Our secondary aim was to detect the optimal cut-off points of UT shear modulus in identifying athletes with rotator cuff tendinopathy. We hypothesized that overhead athletes with rotator cuff tendinopathy would exhibit a higher UT shear modulus than asymptomatic athletes during both active contraction and passive positioning.

## Materials and Methods

### Participants

Forty-three male volleyball players aged between 18 and 35 years (mean age = 22.9±3.5 years) participated in this study. They were recruited from the local sports clubs and universities. They had a training experience of more than three years. Individuals with frozen shoulder (25% limitation of passive shoulder motion in two or more motions), history of shoulder fractures, shoulder instability or dislocation, shoulder surgery or clinical treatment for a shoulder injury, and those who were taking steroids or muscle relaxants were excluded.

Information such as age, gender, height, weight, body mass index (BMI), the number of years in volleyball training, and training hours per week were recorded. Clinical tests were conducted by an experienced physiotherapist to allocate the participants into the rotator cuff tendinopathy group or the asymptomatic group. In the present study, rotator cuff tendinopathy was defined as (1) presence of shoulder pain during training for more than three months [26], (2) had at least three out of five positive tests: painful arc, pain or weakness with resisted external rotation, Neer test, Kennedy-Hawkins test, and Jobe test [27]. The intensity of pain being provoked should be ≥ 3/10 on a visual analogue scale (VAS) [26], and (4) ultrasound image showed the presence of non-homogeneity or partial tear in the supraspinatus tendon [26]. In asymptomatic group, participants had no shoulder pain during volleyball training and clinical tests showed no positive results. The study was approved by the Human Subjects Ethics Sub-committee of the Departmental Research Committee, The Hong Kong Polytechnic University (Reference number: HSEARS20141201002), and all participants gave their written informed consent before the study. All procedures adhered to the Declaration of Helsinki.

### Procedures

**Fig 1:**
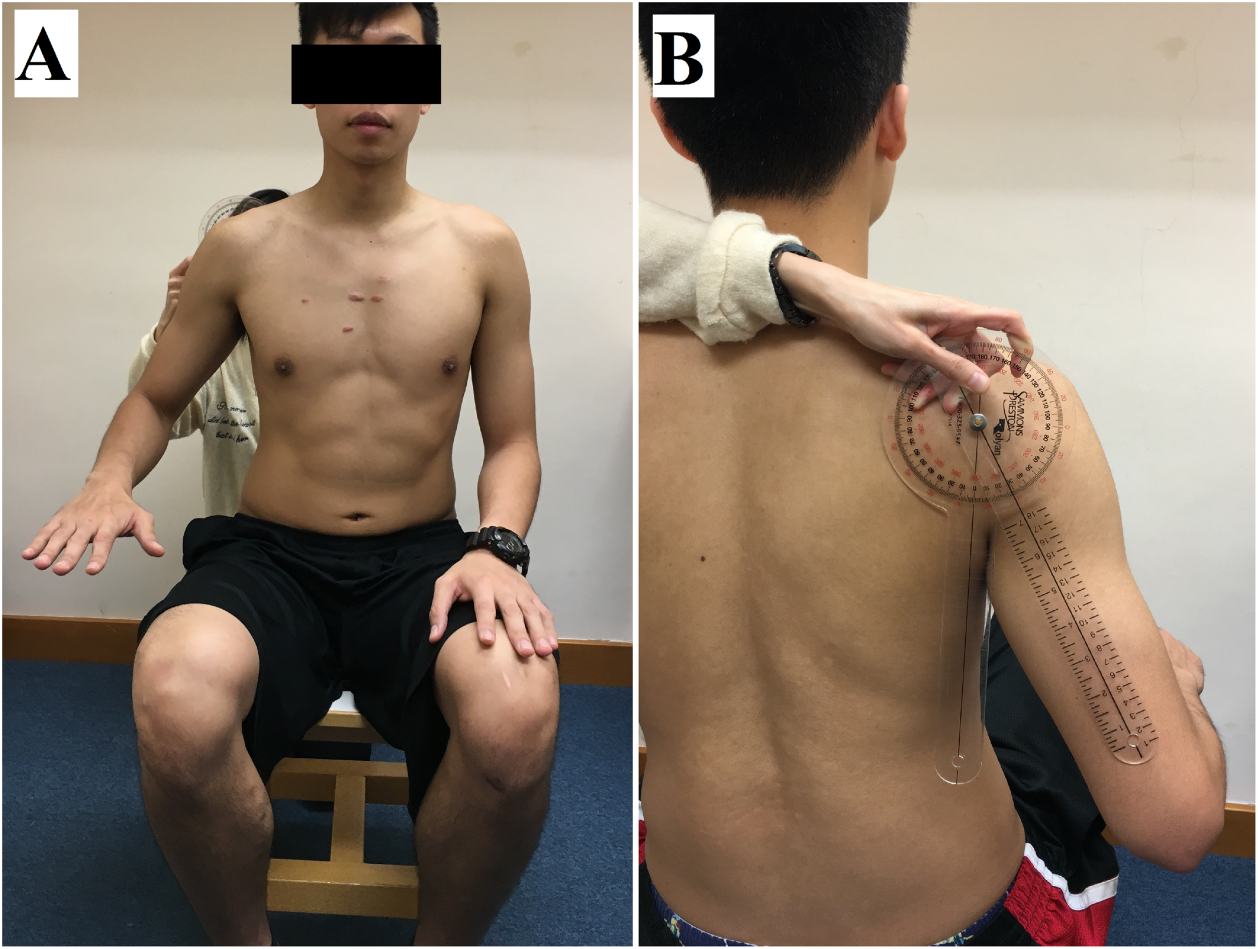
Example of the subject’s position for the measurement of upper trapezius muscle shear modulus during the active task at 30° of shoulder abduction. (A) Front view; (B) Back view.

**Fig 2:**
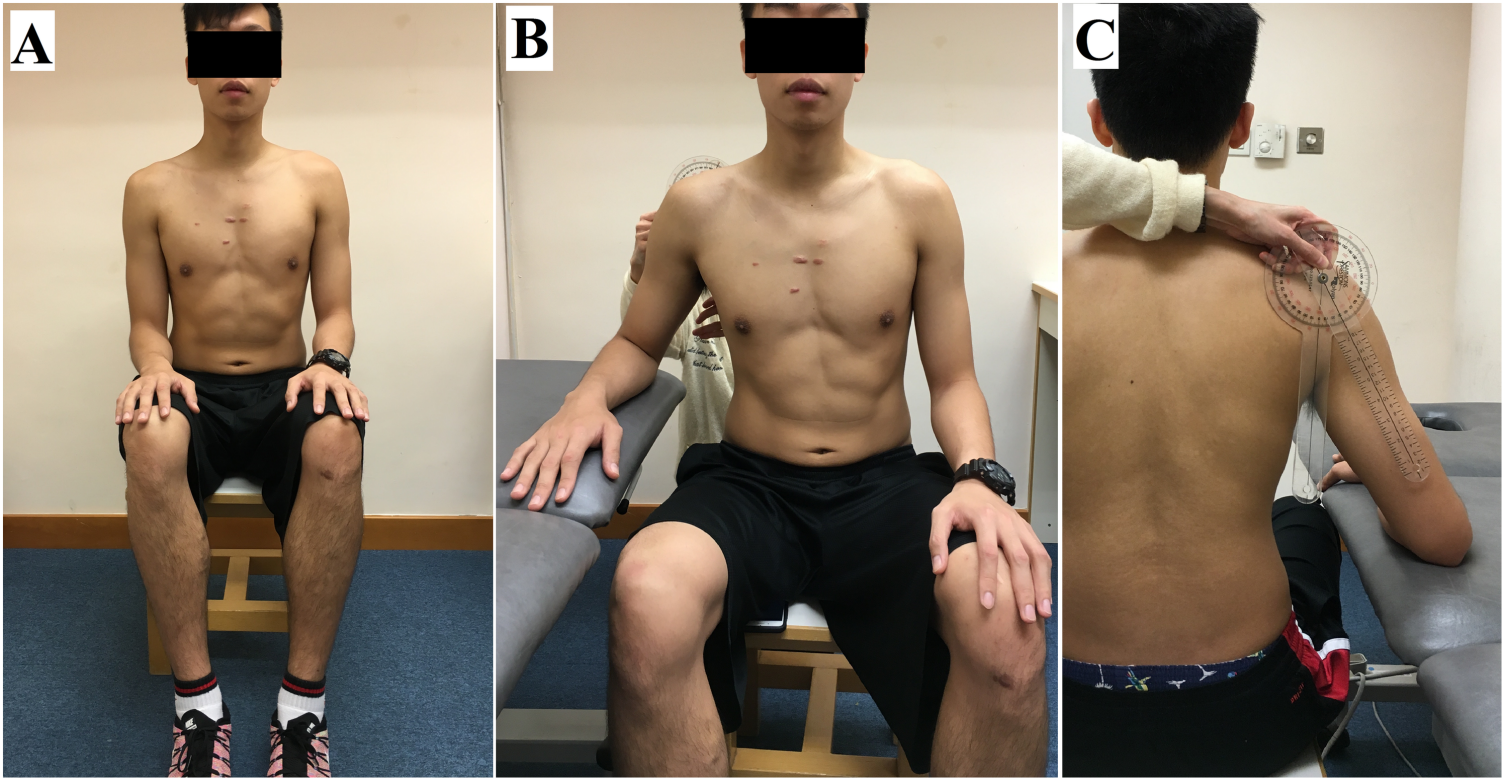
Example of the subject’s position for the measurement of upper trapezius muscle shear modulus during the passive task. (A) 0° of shoulder abduction; (B) 30° of shoulder abduction—front view; (C) 30° of shoulder abduction—back view.

UT shear modulus was measured on the dominant side (defined as the side of the throwing arm) during active arm holding at 30° and 60° of shoulder abduction (active tasks), and after the arm was passively positioned at 0°, 30° and 60° of shoulder abduction (passive tasks). Each participant was asked to sit upright on a stool with the head in neutral position. For the active tasks, the participants were asked to abduct their arm at 30° and 60° for 10 seconds, with elbow flexion at 90° and forearm in pronation (Fig 1). For the passive tasks, the participants were asked to stay fully relax with their elbow flexed at 90° and forearm in pronation. The arm was first rested on hip at 0° of shoulder abduction and was then passively positioned at 30° and 60° of shoulder abduction on a plinth supported (Fig 2). The angle of shoulder abduction was measured by a manual goniometer (Sammons Preston, Royan, Canada). To avoid any influence of muscle fatigue (if any) on the measured parameters, the passive tasks at 0°, 30° and 60° abduction were performed before the active tasks at 30° and 60° abduction. Three elastography measurements were recorded in each arm position/task, and a 1-minute rest was given between each arm position.

### Elastography

**Fig 3:**
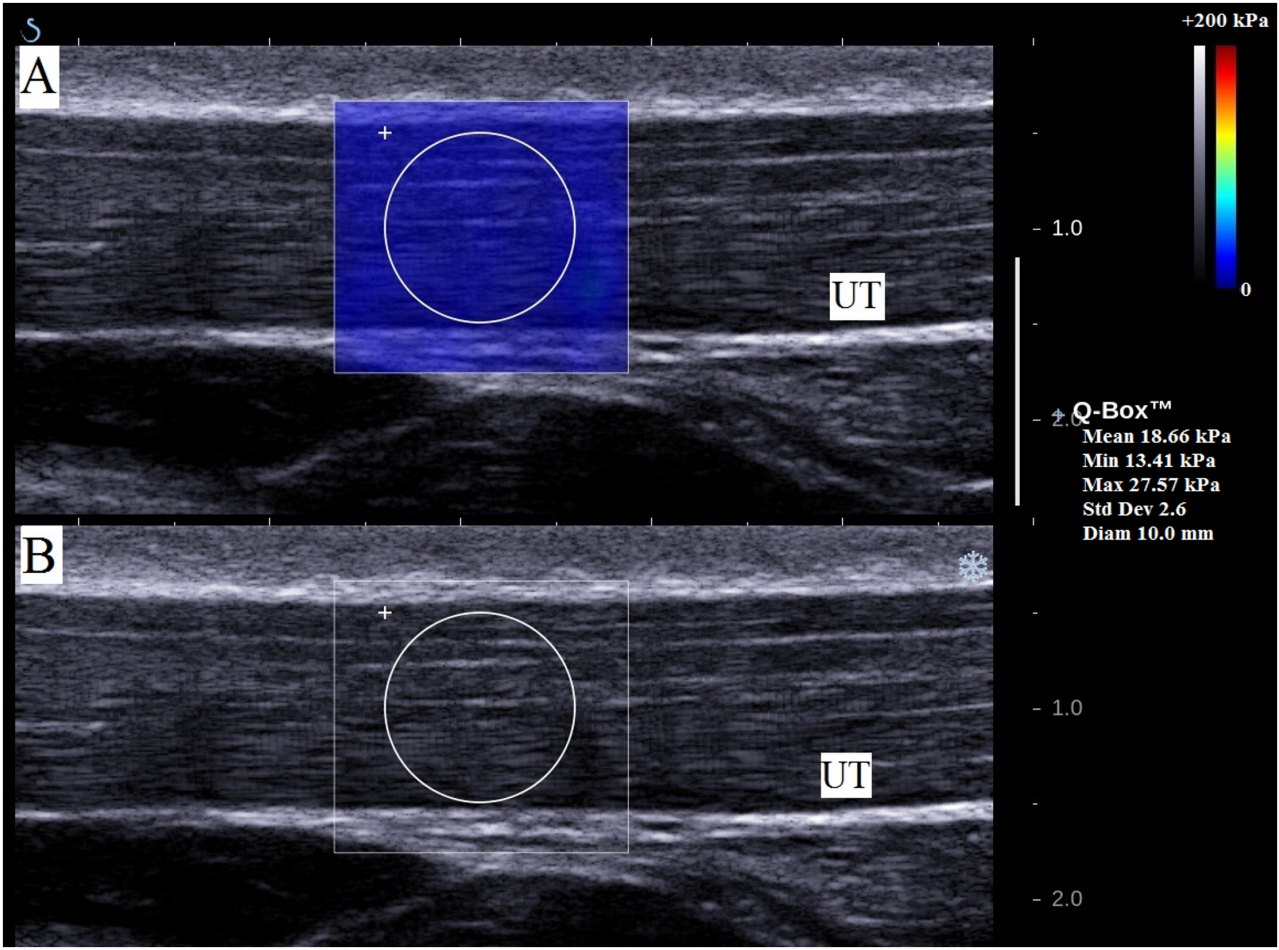
Individual example of a shear modulus map of the upper trapezius (UT) muscle. (A) Color-coded box presentations of the UT elasticity (stiffer areas were coded in red and softer areas in blue) superimposed on a longitudinal grey scale B-mode image. To obtain a representative value, the shear modulus was averaged over a circular area. (B) B-mode image of the UT muscle.

UT shear modulus was measured using an Aixplorer^®^ ultrasound scanner (V4, Supersonic Imagine, Aix-en-Provence, France). The scanner was coupled with a linear transducer array (4-15MHz; SuperLinear 15–4, Vermon, Tours, France) and used in the Shear Wave Elastography mode (general preset). As proposed previously [25], the correct probe location was first determined using B-mode images. Once the muscle was identified, the shear wave elastography mode was turned on. The probe was placed perpendicularly to the skin and parallel to the muscle fibers to minimize muscle anisotropic artefact [28,29]. To avoid tissue deformation, the US transducer was placed very lightly on top of a generous amount of US gel on the surface of the skin [25,30]. The absence of change in muscle thickness was then verified on the B-mode image [30]. Maps of the shear modulus were obtained with a spatial resolution of 1 × 1 mm (Fig 3).

The software of the ultrasound scanner (Supersonic Imagine, France) was used to measure mean shear modulus values across circular regions. The diameter of the circular region was adjusted according to the thickness of the UT muscle (Fig 3) [25,31]. The measurements were averaged over the three measurements per position/task to obtain a representative value of muscle stiffness. This procedure has been shown to provide an excellent within- and between-session repeatability [25]. The Aixplorer scanner provides Young modulus values that require the assumption of an isotropic material. However, skeletal muscles cannot be considered as isotropic materials. Therefore, as classically undertaken in other experiments [32–34], all the measurements were divided by 3 to convert the value to represent the shear modulus.

### Statistical Analysis

Distributions consistently passed the Shapiro-Wilk normality test (all p>0.05). Independent t test was conducted to compare the age, body weight, height, BMI, the number of years in volleyball training, and training hours per week between the two groups (asymptomatic athletes and athletes with rotator cuff tendinopathy).

To address the first aim of this study, repeated measures of ANOVAs were used to determine between-group difference in UT shear modulus at different arm positions (within subject factors: arm position; between-subject factor: group). This was repeated for each task (i.e. passive and active task). When significant interactions were found, independent t-test for comparisons of means for each dependent measure were used and post-hoc analysis was performed using Bonferroni tests to compare the differences between the groups (adjusted *p* values are reported). To address the second aim, receiver operating characteristic (ROC) curves analyses were used to determine a cut-off point of UT shear modulus for both the passive and the active tasks that had significant group difference. Youden’s index (J) was computed from the sensitivity and specificity values (J = Sensitivity + Specificity– 1). The highest index represented the best overall sensitivity and specificity and defined as the cut-off point for identification of subjects with and without rotator cuff tendinopathy [5]. The area under the curve (AUC), the cut-off scores together with the sensitivity and specificity values were reported. The statistical analyses were performed using SPSS Version 23 for Windows (SPSS Inc, Chicago, IL.) The level of significance for all tests was set at 0.05.

## Results

### Demographic data

Among the 43 volleyball players, 26 reported pain or discomfort on the shoulder during training and clinical tests suggested the presence of rotator cuff tendinopathy. Demographic data are depicted in Table 1. No between-group differences were found for any of the tested parameters (all p values >0.08).

**Table 1:** Demographic data of participants.

	Healthy athletes (n = 17)	Athletes with rotator cuff tendinopathy (n = 26)
Age (years)	21.7 ± 3.5	23.6 ± 3.3
Weight (kg)	69.6 ± 5.1	70.0 ± 9.0
Height (cm)	179.4 ± 6.0	178.6 ± 6.9
Body Mass Index (kg/m^2^)	21.7 ± 1.9	21.9 ± 2.1
Years of sports training	8.7 ± 3.7	10.3 ± 3.3
Training hours per week	6.9 ± 2.2	7.1 ± 2.9
Duration of shoulder pain (months)	-	21.9 ± 17.1

Values are mean ± SD

### Active tasks

**Fig 4:**
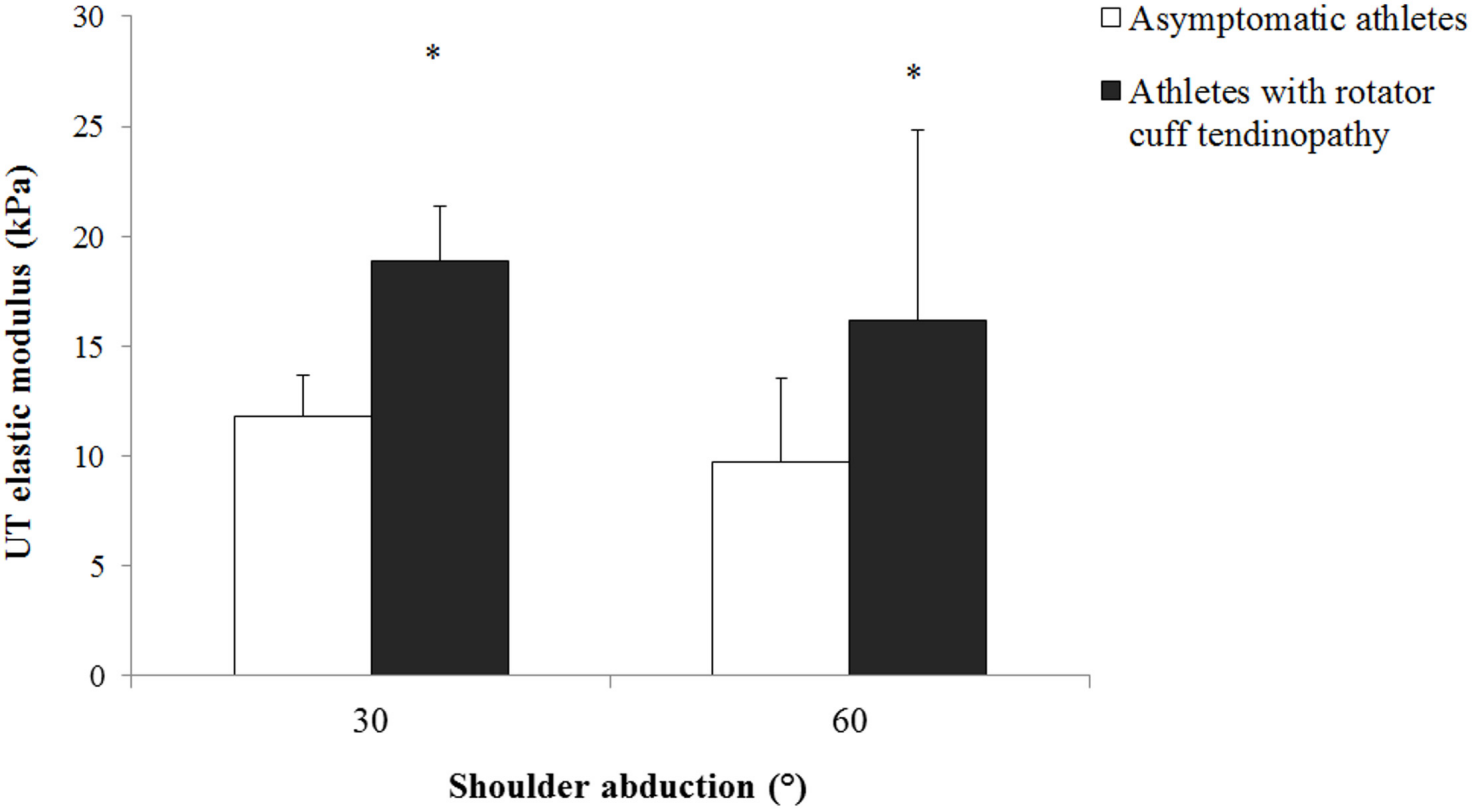
Mean and standard deviation for the upper trapezius shear modulus measured during active arm holding at different abduction angles in asymptomatic athletes (white bar) and athletes with rotator cuff tendinopathy (black bar). * Denotes significant difference between groups (p<0.05).

Although there was no significant arm position × group interaction on the UT shear modulus measured during the active tasks (p = 0.851), there was a significant main effect of group on the UT shear modulus (p = 0.002). Post hoc analysis revealed that the UT shear modulus was higher in athletes with rotator cuff tendinopathy (average between the two positions: 17.1±1.1 kPa) than the asymptomatic athletes (average between the two positions: 10.5±1.6 kPa; p = 0.002), regardless the arm position (Fig 4).

### Passive tasks

**Fig 5:**
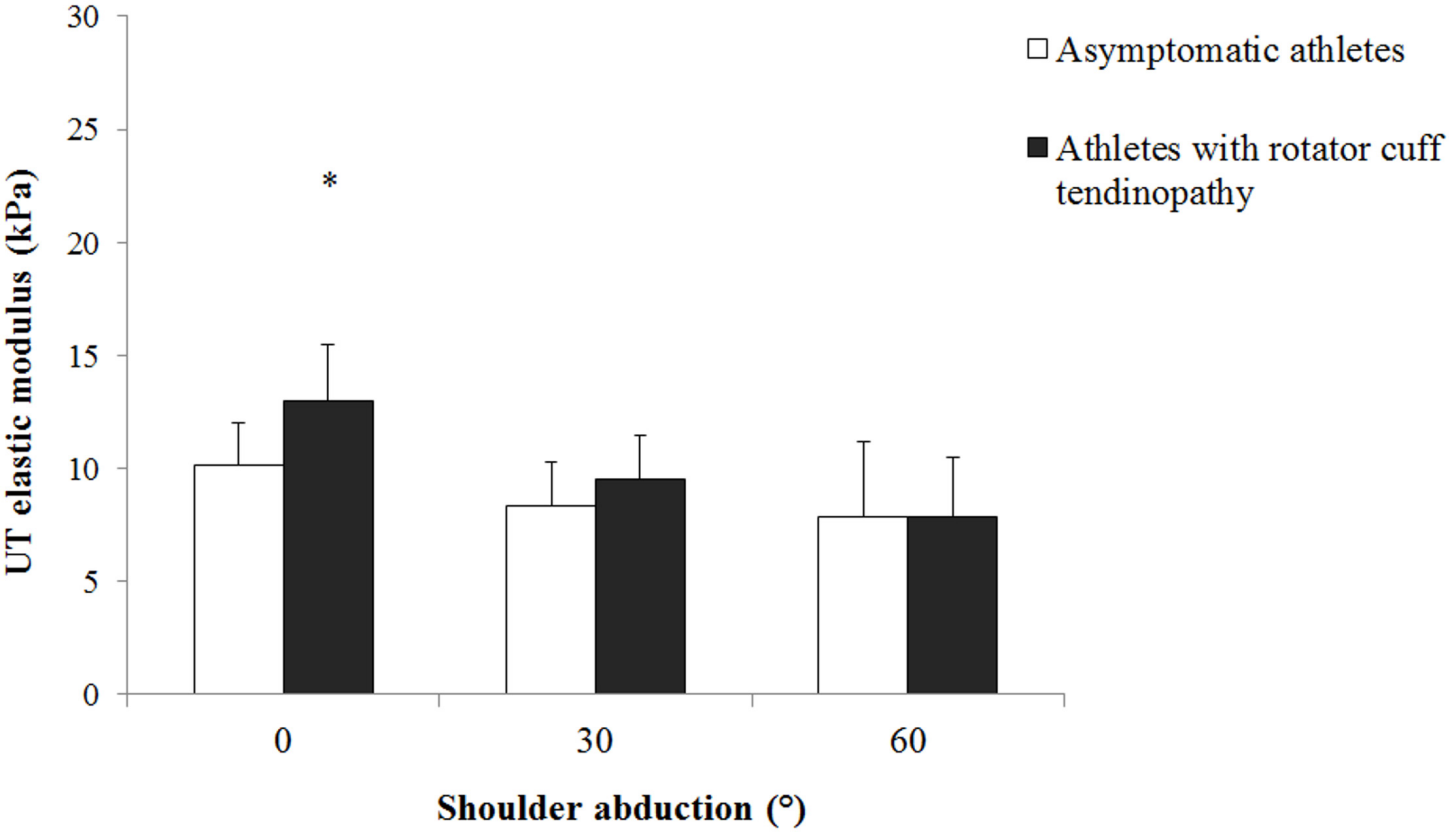
Mean and standard deviation for the upper trapezius shear modulus measured during passive arm positioning at different abduction angles in asymptomatic athletes (white bar) and athletes with rotator cuff tendinopathy (black bar). * Denotes significant difference between groups (p<0.05).

There were a significant effect of group (p = 0.016) and a significant arm position × group interaction (p = 0.016) on the UT shear modulus measured during the passive tasks. Post hoc comparisons revealed that athletes with rotator cuff tendinopathy exhibited higher UT shear modulus than asymptomatic athletes at 0° of shoulder abduction (13.0±2.5 kPa vs 10.2±1.8 kPa, p = 0.001). In addition, there was a trend (p = 0.078) of higher UT shear modulus at 30° of shoulder abduction in athletes with rotator cuff tendinopathy (9.5±1.9 kPa) than asymptomatic athletes (8.3±1.9 kPa) (Fig 5). No between-group differences were found at 60° of shoulder abduction (p = 0.994).

### Optimal cut-off point in identifying overhead athletes with SIS

ROC curves were constructed to determine the optimal cut-off point for UT shear modulus measured during active and passive tasks in identifying athletes with rotator cuff tendinopathy. When considering the active task at 30° and 60° of shoulder abduction, the area under the curve was 0.757 (p = 0.008) and 0.816 (p = 0.002), respectively. Youden’s index showed that a shear modulus of 12.0 kPa at 30° of shoulder abduction yielded a sensitivity of 0.84 and specificity of 0.57; and a shear modulus of 9.5 kPa at 60° of shoulder abduction yielded a sensitivity of 0.88 and a specificity of 0.67 in identifying athletes at risk for rotator cuff tendinopathy. When considering the passive task at 0° of shoulder abduction, a cut-off of 12.2 kPa yielded a sensitivity of 0.73 and specificity of 0.86 (AUC = 0.817, p = 0.001) in identifying athletes at risk for rotator cuff tendinopathy. The AUCs for position at 30° (p = 0.079) and 60° of shoulder abduction (p = 0.938) were not statistically significant and no cut-off point was found.

## Discussion

Taking advantage of elastography, the present study showed that athletes with rotator cuff tendinopathy exhibited higher UT shear modulus during active arm holding than asymptomatic athletes. UT shear modulus was also higher in athletes with rotator cuff tendinopathy than in asymptomatic athletes during the resting arm position at 0° of shoulder abduction. In addition, we determined optimal cutoff points of UT shear modulus during active and passive tasks that can be used for identification of overhead athletes with rotator cuff tendinopathy.

This is the first study to quantify UT shear modulus in overhead athletes with and without rotator cuff tendinopathy at different arm positions. As proposed by recent works [18], we considered muscle shear modulus as an index of muscle stiffness/tension. Athletes with rotator cuff tendinopathy exhibited higher UT shear modulus than the asymptomatic athletes during the active tasks suggesting a higher UT muscle tension during active arm holding. It is important to consider that muscle tension measured during this active task is the combination of both active and passive forces. By measuring the UT shear modulus during passive tasks at the same angles than those used for the active tasks, the present study was able to discriminate between the active and passive mechanisms responsible for a higher UT tension. No between-group difference was found during the passive tasks when the arm was passively positioned at 30° and 60° of shoulder abduction. Consequently, the higher UT shear modulus values measured during the active tasks in athletes with rotator cuff tendinopathy are likely explained by an increased active tension associated to an increased activation rather than intrinsic change in muscle mechanical properties. This is consistent with previous EMG findings that reported an increase in UT EMG amplitude in people with rotator cuff tendinopathy compared to pain-free controls [13–15]. This increased UT activation has clinical consequences such as a decreased activation level and a delayed onset activation of serratus anterior and lower trapezius [35]; and an altered scapular kinematics related to a greater superior translation of the scapula with less efficient upward rotation and posterior tipping [14,15]. These alterations may ultimately compromise the subacromial space and contribute to rotator cuff tendinopathy [11,12]. Even if these results align with previous EMG data, it is important to note that shear modulus measurements have the advantage to not be influenced by cross-talk and may therefore be more sensitive than EMG to detect increased tension. In addition, the difference in passive tension can only be assessed using elastography.

Interestingly, although no between-group difference in UT modulus was observed at 30° and 60° of shoulder abduction during the passive tasks, the athletes with rotator cuff tendinopathy exhibited a significant higher shear modulus than the asymptomatic athletes when the arm was passively positioned at 0° of shoulder abduction. This apparent discrepancy between the arm positions is likely explained by the fact that UT is significantly more stretched at 0° than 30° and 60° of shoulder abduction. This higher passive tension at 0° is confirmed by the higher shear modulus values measured at 0° than 30° and 60° of shoulder abduction (Figs 4 and 5). It is therefore logical that between-group differences in UT shear modulus appear when significant passive tension is produced, i.e. at 0° of shoulder abduction. This passive tension is important to consider as it is related to the muscle extensibility [36]. In this connection, rounded or forward shoulders are common postural deviation in overhead athletes with rotator cuff tendinopathy [37–39], that might associated with alterations in scapular kinematics [15,40,41] and shortening of the pectoralis minor [42]. Kendall et al. [43] stated that individuals with forward shoulder may have stiffer upper trapezius. Higher UT stiffness in athletes with rotator cuff tendinopathy when the arm was passively positioned at 0° of shoulder abduction may be due to postural changed (forward shoulder) in athletes with rotator cuff tendinopathy. Future research is needed to investigate possible relationship between resting UT muscle shear modulus and posture in overhead athletes.

As discussed in the above paragraph, increase in the passive UT muscle shear modulus may be due to postural deviation associated with sports participation. Preventive measures such as postural awareness are essential. In view of its high prevalence of rotator cuff tendinopathy in overhead athletes, early identification of athletes at risk for rotator cuff tendinopathy is important. In this way, we found an optimal cut-off shear modulus of 12.2 kPa at 0° of shoulder abduction yielded a sensitivity of 0.73 and specificity of 0.86 in identifying athletes with cuff tendinopathy. In other words, athletes with UT passive shear modulus greater than 12.2 kPa may have higher risk of developing rotator cuff tendinopathy.

It is difficult to determine from this cross-sectional study whether the observed changes in UT shear modulus are the cause or the consequence of rotator cuff tendinopathy. Changes in UT shear modulus were observed in athletes with rotator cuff tendinopathy during the active tasks, suggesting that changes in active tension of UT may be a consequence of pain associated with rotator cuff tendinopathy. In this way, it is classically thought that the nervous system modifies load on painful tissue to protect this tissue from further pain or injury [44–46]. An increase in active tension of the UT in people with chronic shoulder pain may be an adaptation or compensatory strategy to preserve the subacromial space by elevating the scapular and reduce pain during arm elevation [14,47]. Nevertheless, it should also be noted that an excessive UT tension would alter the normal scapular motion by increasing superior translation of the scapula with less efficient upward rotation and posterior tipping [15,47]. Clinical consequence of this alteration is believed to compromise the subacromial space and may be a potential mechanism to either cause or aggravate impingement symptoms [12,47]. This is in line with a pain theory suggesting that motor adaptations to pain may have short-term benefit with long-term consequences [44,48].

### Limitations

The present study requires consideration of several limitations. First, SSI provides an estimate of the shear modulus of an assumed linear and purely elastic incompressible material. The effects of non-linearity, pre-strain, viscoelasticity and anisotropy are therefore ignored. Assumptions were made about the biomechanical nature of the tissue and this is not meant to represent a ‘‘true” mechanical property but simply a measure in the domain of stiffness/elasticity, which has been proved to be useful clinically [18]. In addition, even though within-subject change in muscle shear modulus can be interpreted as change in muscle tension [18], it is unclear whether the difference in muscle stiffness between individuals can be directly interpreted as a difference in muscle tension. Future studies may use normalization of shear modulus to that measured during a maximal voluntary contraction to provide a more direct comparison of muscle tension between individuals. In this experiment, the saturation limit of the elastography scanner did not allow us to perform such a normalization procedure. Second, it is important to consider that to understand the biomechanical consequences of this increased UT shear modulus on the shoulder joint, we need to consider the force-generating capacity of the UT muscle. This capacity is mainly determined by its physiological-cross sectional area and its level arm. Future studies need to consider these parameters. Third, EMG was not used to confirm the absence of muscle activation during the passive tests. However, participants were asked to relax and no sign of muscle contraction was visible on the B-mode image. Together, it makes us confident that participants remained in a passive state while their arms were fully supported.

## Conclusions

The present study showed that athletes with rotator cuff tendinopathy exhibited higher UT shear modulus during active arm holding than the asymptomatic athletes. UT shear modulus was also higher in athletes with rotator cuff tendinopathy than in the asymptomatic athletes during the resting arm position at 0° of shoulder abduction. Optimal cut-off points of UT shear modulus during active and passive tasks that can be used for identification of overhead athletes with rotator cuff tendinopathy. Such findings suggest that monitoring and maintenance of passive and active UT muscle stiffness/tension are essential for the prevention/rehabilitation for rotator cuff tendinopathy.
